# Ribosomes: the future of targeted therapies?

**DOI:** 10.18632/oncotarget.1511

**Published:** 2013-10-17

**Authors:** Virginie Marcel, Frédéric Catez, Jean-Jacques Diaz

**Affiliations:** Centre de Recherche en Cancérologie de Lyon, UMR Inserm 1052 CNRS 5286, Centre Léon Bérard, Lyon, France and Université de Lyon, Lyon, France

Ribosomes are ribo-nucleoprotein complexes that read mRNA to synthesize protein during translation. It is firmly established that the amount of ribosomes strongly correlates with the rate of protein synthesis and with cell growth and proliferation. Because production of ribosomes is a high energy-consuming task, ribosome biogenesis must be finely tuned to adapt the pool of functional ribosomes to the cell's needs at a given time for a defined environment. Signalling, via nutrient availability and growth factors, controls ribosomal RNA (rRNA) transcription through regulation of RNA polymerase I activity. Compared to healthy cells, cancer cells demand a global increase in protein synthesis to support their hyper-proliferative behaviour. In cancer cells, activation of growth signalling pathways and oncogenes or inactivation of tumour suppressors, allows production of large amounts of ribosomes by increasing RNA polymerase I activity. Although this observation suggests that ribosome production could be targeted for anti-cancer drug development, the first proof-of-principle that specific inhibition of ribosome biogenesis is a potent anti-cancer therapy has been reported only recently. Using a cell-based screen assay, Drygin and collaborators identified the small molecule CX-5461 as a selective inhibitor of RNA polymerase I activity [1]. Later, Bywater and collaborators reported that CX-5461 specifically kills B lymphoma cells in Eμ-MYC murine models through a non-genotoxic activation of p53, without affecting wild-type B cells [2]. In addition, this treatment results in disease remission with significantly improved outcomes. A phase I clinical trial of CX-5461 has been recently initiated in patients with haematological cancers to determine the effectiveness of specific inhibition of RNA polymerase I activity as a targeted therapeutic approach to fight cancer.

In addition to “quantitative” variations of ribosomes, “qualitative” alterations of ribosomes have been reported in some pathologies. Changes in ribosome composition, including changes in ribosomal proteins or in rRNA chemical modifications, are associated with different familial syndromes, globally named ribosomopathies (e.g. Diamond-Blackfan anemia, 5q Syndrome). Among them, Dyskeratosis congenita which is characterized by premature aging and increase in cancer susceptibility, is associated with expression of mutant DKC1 enzyme that is involved in pseudo-uridylation of rRNA [3]. Mutant DKC1 modifies the rRNA pseudo-uridylation pattern of ribosomes and reduces translational efficiency of a distinct subset of mRNAs that are at the origin of the pathology and of cancer susceptibility. Indeed, the catalytic activity of ribosomes is driven by rRNA, whose dynamic changes of structure are involved in both mRNA decoding and formation of peptide bonds. rRNA chemical modifications, which include pseudo-uridylation and ribose methylation, are essential for maintaining rRNA structures as well as RNA:RNA and RNA:protein interactions, and it has been shown that their alteration directly affects intrinsic ribosome activity during translation.

We recently reported that rRNA methylation pattern is altered in mammary cellular models that mimic tumour progression [4, 5]. Alteration of rRNA methylation pattern occurs in cells carrying an inactive p53 protein, which is mutated in almost 25% of breast cancer tumours [5]. Indeed, we showed that mutations in the *TP53* gene counteract the p53-mediated repression of fibrillarin (FBL), the 2'-O-methyltransferase of the rRNA. In breast cancer tumours, high expression of FBL is an independent marker of poor patient outcome. The clinical value of FBL and the FBL-induced oncogenic properties of cells might result, at least in part, from the impact of altered rRNA methylation pattern on the intrinsic activity of the ribosome. Indeed, changes in rRNA methylation are associated with increased translation of a specific subset of mRNA encoding key oncoproteins and also with a decrease in translational fidelity. In particular, we observed that p53-mediated alteration of rRNA methylation increased amino-acid mis-incorporation and stop codon readthrough.

Based on these observations, we can speculate that specifically targeting activity of altered ribosomes produced in cancer cells may constitute a novel anti-cancer therapy. Several small molecules have been developed that affect the intrinsic activity of ribosomes through modulation of rRNA structural properties, such as aminoglycosides [6]. Considering that the intrinsic activity of ribosomes is driven by rRNA and that rRNA composition of ribosomes is modified in cancer cells, we anticipate that small molecules drugs specifically inhibiting the intrinsic activity of altered ribosomes produced in cancer cells will be developed in the near future. Alternatively, components of ribosome biogenesis involved in rRNA maturation could be targeted, such as FBL. Since FBL over-expression is associated with acquisition of proliferative capacities in both an anchorage-dependent and -independent manner and also in resistance to chemotherapy [5], inhibition of FBL might limit tumour progression. A recent study supports this notion, since knockdown expression of FBL reduces tumour development in mammary xenograft models by promoting p53-dependent G1 arrest [7].

After 60 years of research dedicated to ribosomes, it now appears that ribosomes represent a powerful target in anti-cancer therapy. Both the inhibition of ribosome biogenesis, and the targeting of the altered ribosomes produced by cancer cells may provide an efficient and selective approach for killing tumor cells. Cellular translation is receiving increasing attention in cancer-therapy, with translation regulatory factors as targets [8]. While the ribosome emerges as a major player in translational regulation, its place in the list of potential therapeutic target is reinforced. A greater understanding of the impact of rRNA chemical modifications on intrinsic activity of ribosomes and on translational regulation and tumor phenotype will open new avenues to exploit ribosomes in targeted therapies.
